# Mitochondria Dysfunction-Mediated Molecular Subtypes and Gene Prognostic Index for Prostate Cancer Patients Undergoing Radical Prostatectomy or Radiotherapy

**DOI:** 10.3389/fonc.2022.858479

**Published:** 2022-04-06

**Authors:** Dechao Feng, Xu Shi, Facai Zhang, Qiao Xiong, Qiang Wei, Lu Yang

**Affiliations:** Department of Urology, Institute of Urology, West China Hospital, Sichuan University, Chengdu, China

**Keywords:** molecular subtype, prostate cancer, mitochondria dysfunction, biochemical recurrence, radical prostatectomy, radical radiotherapy

## Abstract

**Background:**

Given the age relevance of prostate cancer (PCa) and the role of mitochondrial dysfunction (MIDS) in aging, we orchestrated molecular subtypes and identified key genes for PCa from the perspective of MIDS.

**Methods:**

Cluster analysis, COX regression analysis, function analysis, and tumor immune environment were conducted. We performed all analyses using software R 3.6.3 and its suitable packages.

**Results:**

CXCL14, SFRP4, and CD38 were eventually identified to classify the PCa patients in The Cancer Genome Atlas (TCGA) database and the Gene Expression Omnibus (GEO) dataset into two distinct clusters. Patients in the cluster 2 had shorter BCR-free survival than those in the cluster 1 in terms of both TCGA database and GEO dataset. We divided the patients from the TCGA database and the GEO dataset into high- and low-risk groups according to the median of MIDS-related genetic prognostic index. For patients in the TCGA database, the biochemical recurrence (BCR) risk in high-risk group was 2.34 times higher than that in low-risk group. Similarly, for patients in the GEO dataset, the risk of BCR and metastasis in high-risk group was 2.35 and 3.04 times higher than that in low-risk group, respectively. Cluster 2 was closely associated with advanced T stage and higher Gleason score for patients undergoing radical prostatectomy or radiotherapy. For patients undergoing radical prostatectomy, the number of CD8^+^ T cells was significantly lower in cluster 2 than in cluster 1, while cluster 2 had significantly higher stromal score than cluster 1. For patients undergoing radical radiotherapy, cluster 2 had significantly higher level of CD8^+^ T cells, neutrophils, macrophages, dendritic cells, stromal score, immune score, and estimate score, but showed lower level of tumor purity than cluster 1.

**Conclusions:**

We proposed distinctly prognosis-related molecular subtypes at genetic level and related formula for PCa patients undergoing radical prostatectomy or radiotherapy, mainly to provide a roadmap for precision medicine.

## Introduction

Prostate cancer (PCa) is the most common non-skin malignant tumor diagnosed among American men in 2021, accounting for 26% (1). For localized PCa, radical radiotherapy and radical prostatectomy are the preferred treatment options. However, three-quarters of men will experience biochemical recurrence (BCR) after receiving radical treatment without evidence of overt metastatic disease (2). There has been no agreement on the definition of BCR (3). However, for recurrence patients, the median time to metastasis is 8 years, and the median time from metastasis to death is 5 years (4). Due to the lack of prospective randomized trials with a high level of evidence, the best management for BCR has not yet been confirmed since no intervention is currently considered to extend survival, which highlights the importance of personalized therapy and deciding when to start which treatment.

Very little has been known about the cause of PCa, among which aging is the only definite risk factor (1). Cellular senescence is a driver of aging and age-related diseases. The increase of age is accompanied by the accumulation of senescent cells in the tissues and the appearance of cellular senescence (5). Cell senescence is a cellular stress response caused by irradiation and other macromolecular damage which was once considered to be a tumor suppressor mechanism, but recent studies have shown that senescent cells are metabolically active, and the inflammatory mediators they secrete are called senescence-associated secretory phenotype (SASP) or senescence messaging secretome (6). Senescent cells exacerbate inflammation through SASP, which is called “inflammageing” (7, 8).

Mitochondria have been identified as one of the key regulators of the development of aging phenotypes, especially the pro-inflammatory SASP (9). The role of mitochondria in PCa has gradually become clear with a large number of studies on various nuclear-encoded pathways. There is considerable crosstalk between the nucleus and mitochondria through the retrograde signal from the mitochondria to the nucleus and the anterograde signal from the nucleus to the mitochondria through the translocation of cytoplasmic translation proteins to the mitochondria (10). Mitochondrial damage has been shown to be involved in the pathophysiology of PCa (11), which is a highly hereditary disease (12). Changes in the mitochondrial genome have been proven to be related to predictors of tumor proliferation, metastasis, and BCR (11). Next-generation sequencing of mitochondrial DNA from 115 men showed a positive correlation between the total burden of acquired mitochondrial DNA variants and the elevated Gleason score at diagnosis and BCR (13). Given the age relevance of PCa and the role of mitochondrial dysfunction (MIDS) in aging, we orchestrated molecular subtypes and identified key genes for PCa from the perspective of MIDS, so as to provide a roadmap for the evolution of precision medicine. In addition, we also developed an independent genetic prognosis index to quantify the recurrence risk of patients. Our study has been registered in the ISRCTN registry (No. ISRCTN11560295).

## Methods

### Data Preparation

For the combination of GSE46602 (14), GSE32571 (15), GSE62872 (16), and GSE116918 (17) from the Gene Expression Omnibus (GEO) datasets (18), R package “inSilicoMerging” (19) was used and “removeBatchEffect” function of the “limma (version 3.42.2)” package was used to remove the batch effects (**Supplementary Figure 1**). Subsequently, we extracted the differentially expressed mRNAs between tumor and normal tissues from the GSE46602 (14), GSE32571 (15), and GSE62872 (16), and further conducted the prognosis analysis through log-rank test using the GSE116918 (17). Similar methods were used to proceed the PCa data from the TCGA database in the UCSC XENA (20). Differentially expressed genes (DEGs) were defined as llogFCl ≥0.4 and p.adj. <0.05. P-value of BCR-free survival or metastasis-free survival was restricted to less than 0.05. MIDS-related genes were obtained from the GeneCards (21). The candidate genes were identified through the intersection of DEGs and prognosis-related genes in the GEO and TCGA databases, and the MIDS-related genes. The gene interactions and drug analysis of the candidate genes were performed through the STRING database (22) and GSCALite (23) which included drug data of the cancer therapeutics response portal (CTRP) and genomics of drug sensitivity in cancer (GDSC).

### Molecular Subtypes and Genetic Prognosis Index

R packages “ConsensusClusterPlus” and “limma” were used to subtyping the patients who underwent radical prostatectomy in the TCGA database or underwent radical radiotherapy in the GSE116918 (17) through the three candidate genes. The consensus matrix k value denoted the number of clusters. Subsequently, we analyzed the correlations between the clinical parameters and two clusters and prognostic value of the clusters for PCa patients from the TCGA database and GSE116918 (17). Gene set enrichment analysis (GSEA) of the two clusters was conducted, and p-value of <0.05 and a false discovery rate (FDR) of <0.25 were considered statistically significant (24, 25). Besides, we constructed a MIDS-related genetic prognostic index (MDGPI) according to the results of multivariate COX regression analysis for PC patients in the TCGA database to quantify the BCR risk of patients. The MDGPI formula was as follows: risk score = −1.601 + 0.063 ∗ CXCL14 + 0.176 − SFRP4 − 0.095 ∗ CD38. Then, we used the 248 tumor patients in the GSE116918 (17) to confirm the prognostic value of the MDGPI score.

### Tumor Immune Microenvironment (TME) and Checkpoints

**Figure 1:**
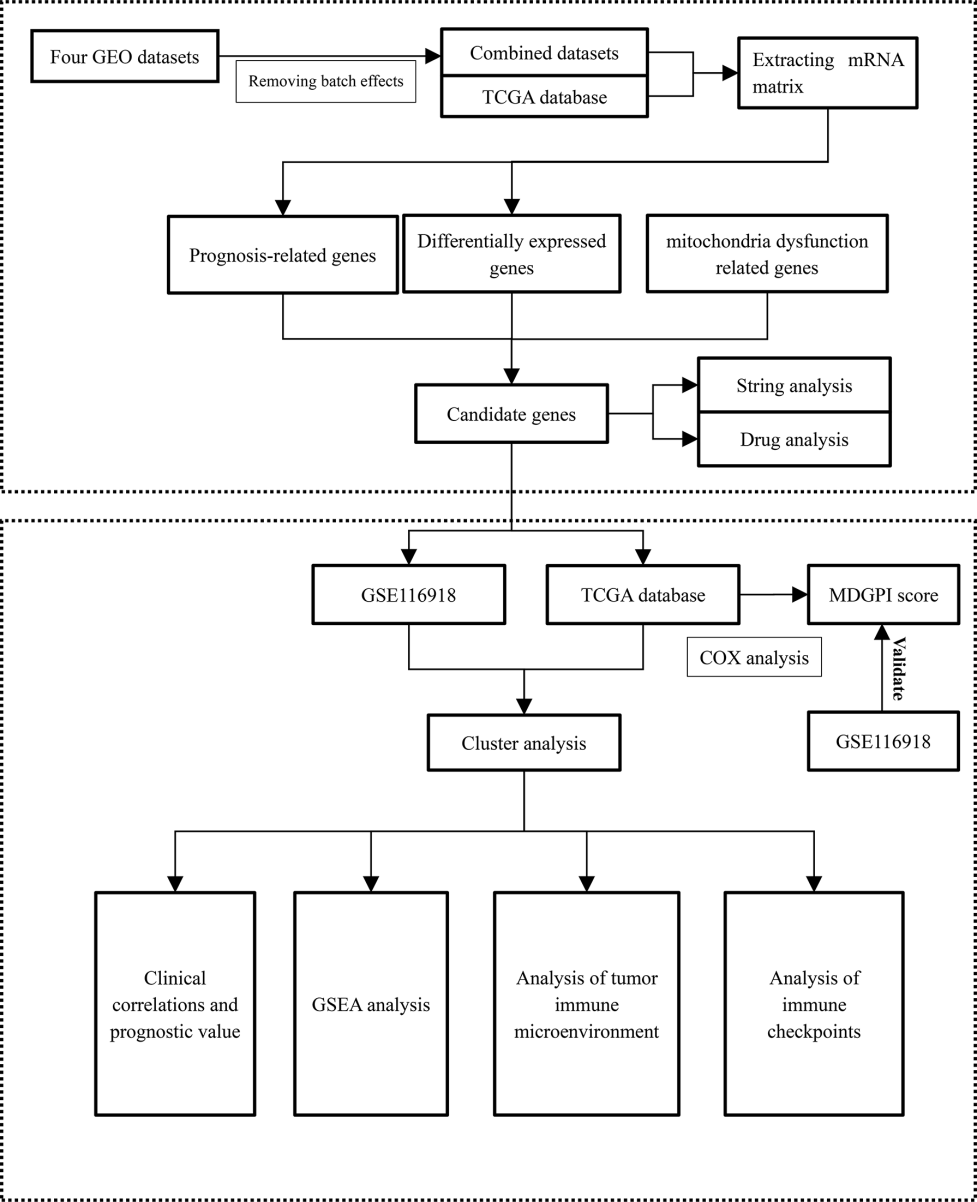
The flowchart of this study. GSEA, gene set enrichment analysis; MDGPI, mitochondrial dysfunction genetic prognostic index; GEO, Gene Expression Omnibus; mRNA, messenger RNA.

We analyzed the tumor immune microenvironment (TME) through the TIMER and ESTIMATE algorithms (26, 27). In addition, 54 and 47 common immune checkpoints were analyzed for PCa patients from the TCGA and GEO databases, respectively. Comparisons between TME components and immune checkpoints and the two clusters were performed through the Wilcoxon rank sum test. The Spearman analysis was used to explore the relationship between MDGPI and TME components and immune checkpoints. Immune checkpoints, which were differentially expressed between the two clusters and were significantly associated with the BCR-free survival for patients in the TCGA database and GSE116918 (17), were identified as well. We presented the flowchart of this study in **Figure 1**.

### Statistical Analysis

We performed all analyses using software R 3.6.3 and its suitable packages. We utilized Wilcoxon test under the circumstance of non-normal data distribution. Variables could be entered into multivariate COX regression analysis if p-value <0.1 in the univariable Cox regression analysis. Survival analysis was conducted through log-rank test and presented as Kaplan–Meier curve. Besides, the Spearman analysis was used to assess the correlations among continuous variables if they did not meet Shapiro–Wilk normality test. Statistical significance was set as two-sided p <0.05. Significant marks were as follows: ns, p ≥0.05; *, p <0.05; **, p <0.01; ***, p <0.001.

## Results

### Molecular Subtype and its Clinical Values

**Figure 2:**
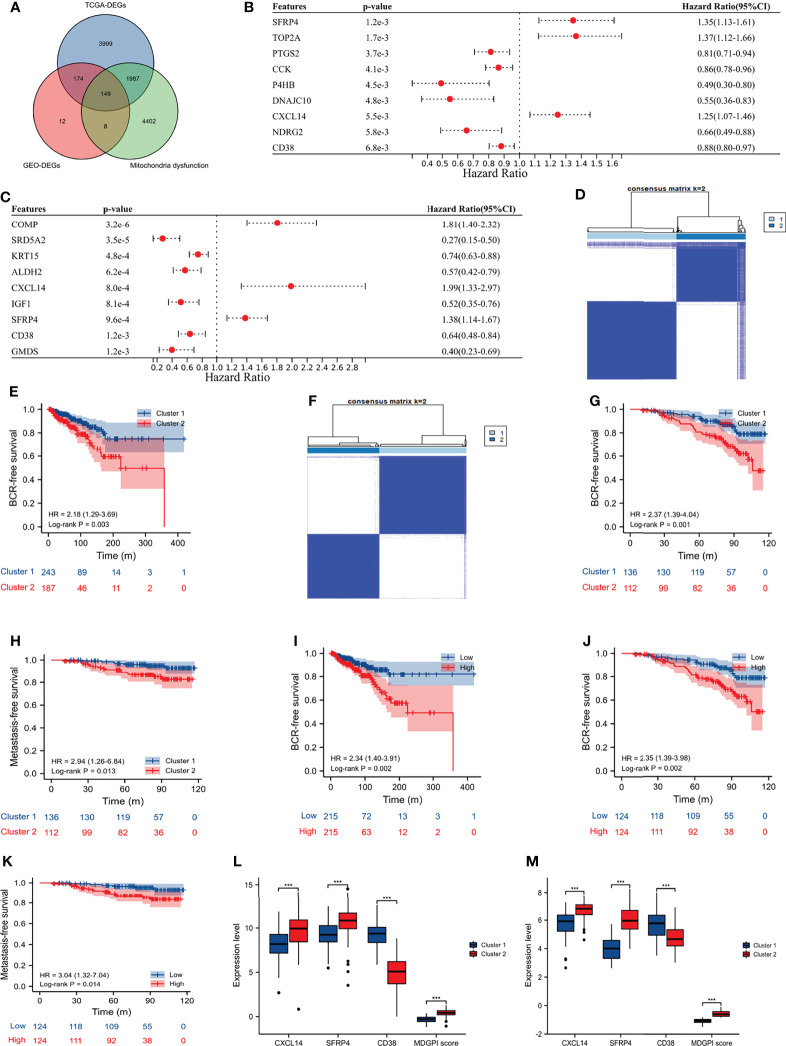
Molecular subtype and MDGPI score. **(A)** Venn plot showing the interaction of differentially expressed genes between tumor and normal samples in the TCGA and GEO datasets, and mitochondrial dysfunction-related genes; **(B)** forest plot showing genes associated with BCR-free survival in the TCGA database; **(C)** forest plot showing genes associated with BCR-free survival in the GSE116918 (17); **(D)** cluster plot showing distinct two groups in the TCGA database; **(E)** Kaplan–Meier curve presenting the BCR-free survival difference of the two clusters in the TCGA database; **(F)** cluster plot showing distinct two groups in the GSE116918 (17); **(G)** Kaplan–Meier curve presenting the BCR-free survival difference of the two clusters in the GSE116918 (17); **(H)** Kaplan–Meier curve presenting the metastasis-free survival difference of the two clusters in the GSE116918 (17); **(I)** Kaplan–Meier curve presenting the BCR-free survival difference of the high- and low-risk groups based on the median of MDGPI in the TCGA database; **(J)** Kaplan–Meier curve presenting the BCR-free survival difference of the high- and low-risk groups based on the median of MDGPI in the GSE116918 (17); **(K)** Kaplan–Meier curve presenting the metastasis-free survival difference of the high- and low-risk groups based on the median of MDGPI in the GSE116918 (17); **(L)** comparisons between cluster 2 and cluster 1 in the TCGA database for CXCL14, SFRP4, CD38, and MDGPI score; **(M)** comparisons between cluster 2 and cluster 1 in the GSE116918 (17) for CXCL14, SFRP4, CD38, and MDGPI score. BCR, biochemical recurrence; MDGPI, mitochondrial dysfunction-related genetic prognostic index. ***, p <0.001.

The GSE46602 (14), GSE32571 (15), and GSE62872 (16) had 209 normal and 360 tumor samples, and the GSE116918 (17) contained 248 PCa patients undergoing radical radiotherapy with complete data of BCR and metastasis. Besides, we also obtained 498 tumor and 52 normal samples of PCa from the TCGA database, among which 430 PCa patients undergoing radical prostatectomy had complete data of BCR. After the intersection of DEGs and prognosis-related genes in the GEO and TCGA databases, and the MIDS-related genes (**Figures 2A–C**), CXCL14, SFRP4, and CD38 were eventually identified to classify the PCa patients in the TCGA database into two distinct clusters (**Figure 2D**; consensus matrix k = 2). Moreover, patients in cluster 2 had shorter BCR-free survival than those in cluster 1 (HR: 2.18, 95% CI: 1.29–3.69, p = 0.003; **Figure 2E**). Similarly, we observed that these three genes could obviously distinguish cluster 2 from cluster 1 for patients undergoing radical radiotherapy in the GSE116918 (17) (**Figure 2F**; consensus matrix k = 2), and patients in cluster 2 were more prone to BCR (HR: 2.37, 95% CI: 1.39–4.04, p = 0.001; **Figure 2G**) and metastasis (HR: 2.94, 95% CI: 1.26–6.84, p = 0.013; **Figure 2H**) than their counterparts. We divided the patients from the TCGA database and the GSE116918 (17) into high- and low-risk groups according to the median of MDGPI score. For patients in the TCGA database, the BCR risk in high-risk group was 2.34 times higher than that in low-risk group (95% CI: 1.40–3.91; **Figure 2I**). Similarly, for patients in the GSE116918 (17), the risk of BCR and metastasis in high-risk group was 2.35 and 3.04 times higher than that in low-risk group, respectively (**Figures 2J, K**). In addition, patients in cluster 2 had significantly higher levels of CXCL14, SFRP4, and MGPI score, and lower level of CD38 than those in cluster 1 for PCa patients from the TCGA database (**Figure 2L**) and GSE116918 (17) (**Figure 2M**).

**Figure 3:**
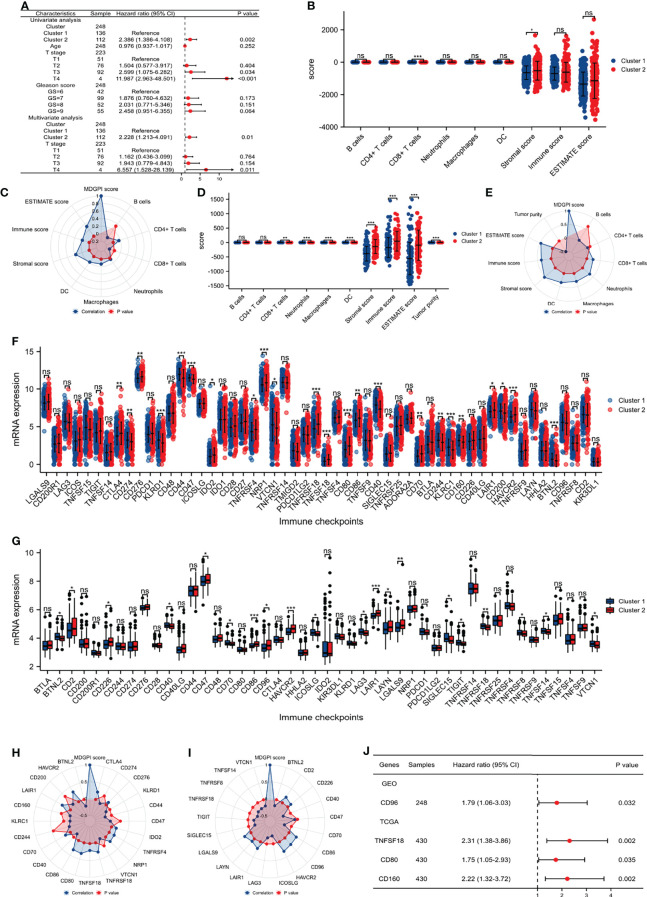
Tumor immune microenvironment and checkpoints analysis. **(A)** COX regression analysis showing the results of clusters and other clinical parameters in the GSE116918 (17); **(B)** comparison between the two clusters for immune cells in the TCGA database; **(C)** radar plot showing the correlations between MDGPI score and immune cells in the TCGA database; **(D)** comparison between the two clusters for immune cells in the GSE116918 (17); **(E)** radar plot showing the correlations between MDGPI score and immune cells in the GSE116918 (17); **(F)** comparison between the two clusters for immune checkpoints in the TCGA database; **(G)** comparison between the two clusters for immune checkpoints in the GSE116918 (17); **(H)** radar plot showing the correlations between MDGPI score and immune checkpoints in the TCGA database; **(I)** radar plot showing the correlations between MDGPI score and immune checkpoints in the GSE116918 (17); **(J)** forest plot showing checkpoints associated with BCR-free survival in the TCGA database and GSE116918 (17). BCR, biochemical recurrence; MDGPI, mitochondrial dysfunction-related genetic prognostic index. ns, p ≥0.05; *, p <0.05; **, p <0.01; ***, p <0.001.

For patients undergoing radical prostatectomy, we found that cluster 2 was significantly associated with older age, BCR, higher N stage, positive residual tumor, higher Gleason score, and advanced T stage (**Table 1**). Similarly, for patients undergoing radical radiotherapy, we observed that cluster 2 was significantly related to BCR, metastasis, higher Gleason score, and advanced T stage (**Table 2**). One rather interesting outcome was that cluster 2 was an independent risk factor for patients undergoing radical radiotherapy (**Figure 3A**).

**Table 1:** The correlations between clinical indicators and clusters in the TCGA database.

Characteristic	Cluster 1	Cluster 2	P-value
Samples (n)	243	187	
Age, median (IQR)	61 (56, 65)	63 (57, 67)	0.010
Biochemical recurrence, n (%)			0.018
No	219 (50.9%)	153 (35.6%)	
Yes	24 (5.6%)	34 (7.9%)	
N stage, n (%)			<0.001
N0	182 (48.5%)	124 (33.1%)	
N1	23 (6.1%)	46 (12.3%)	
Residual tumor, n (%)			0.003
No	170 (40.6%)	103 (24.6%)	
Yes	68 (16.2%)	78 (18.6%)	
Gleason score (GS), n (%)			<0.001
GS = 6	30 (7%)	9 (2.1%)	
GS = 7	149 (34.7%)	57 (13.3%)	
GS = 8	29 (6.7%)	30 (7%)	
GS = 9	35 (8.1%)	91 (21.2%)	
T stage, n (%)			<0.001
T2	111 (26.2%)	44 (10.4%)	
T3	128 (30.2%)	133 (31.4%)	
T4	0 (0%)	8 (1.9%)	

IQR, interquartile range; GS, Gleason score.

**Table 2:** The correlations between clinical indicators and clusters in the GSE116918 (17).

Characteristic	Cluster 1	Cluster 2	P-value
Samples (n)	136	112	
Age, median (IQR)	67 (64, 72)	69 (62, 73)	0.632
T stage, n (%)			<0.001
T1	39 (17.5%)	12 (5.4%)	
T2	42 (18.8%)	34 (15.2%)	
T3	41 (18.4%)	51 (22.9%)	
T4	0 (0%)	4 (1.8%)	
Gleason score (GS), n (%)			<0.001
GS = 6	37 (14.9%)	5 (2%)	
GS = 7	60 (24.2%)	39 (15.7%)	
GS = 8	26 (10.5%)	26 (10.5%)	
GS = 9	13 (5.2%)	42 (16.9%)	
Biochemical recurrence, n (%)			0.005
No	115 (46.4%)	77 (31%)	
Yes	21 (8.5%)	35 (14.1%)	
Metastasis, n (%)			0.041
No	129 (52%)	97 (39.1%)	
Yes	7 (2.8%)	15 (6%)	

IQR, interquartile range; GS, Gleason score.

### TME and Immune Checkpoints Analysis

For patients undergoing radical prostatectomy, the number of CD8^+^ T cells was significantly lower in cluster 2 than cluster 1 (p <0.001), while cluster 2 had significantly higher stromal score than cluster 1 (**Figure 3B**). Moreover, MDGPI score was closely associated with CD4^+^ T cells (r: 0.16), CD8^+^ T cells (r: −0.1), macrophages (r: 0.13), dendritic cells (r: 0.18), stromal score (r: 0.37), immune score (r: 0.19), and estimate score (r: 0.32) (**Figure 3C**). For patients undergoing radical radiotherapy, cluster 2 had significantly higher level of CD8^+^ T cells (p = 0.002), neutrophils (p <0.001), macrophages (p <0.001), dendritic cells (p <0.001), stromal score (p <0.001), immune score (p <0.001), and estimate score (p <0.001), but showed lower level of tumor purity than cluster 1 (p <0.001) (**Figure 3D**). In addition, MDGPI score showed significantly correlations with CD8^+^ T cells (r: 0.23), neutrophils (r: 0.35), macrophages (r: 0.31), dendritic cells (r: 0.35), stromal score (r: 0.53), immune score (r: 0.36), estimate score (r: 0.47), and tumor purity (r: −0.47) (**Figure 3E**).

In terms of immune checkpoints, 23 and 18 checkpoints were significantly differentially expressed between cluster 2 and cluster 1 for PCa patients from the TCGA database (**Figure 3F**) and the GSE116918 (17) (**Figure 3G**), respectively. For patients from the TCGA database, MDGPI was highly associated with CTLA4 (r: 0.23), CD276 (r: 0.12), KLRD1 (r: −0.11), CD44 (r: −0.19), IDO2 (r: 0.15), TNFRSF4 (r: 0.17), NRP1 (r: 0.19), TNFRSF18 (r: 0.28), TNFSF18 (r: 0.21), CD80 (r: 0.33), CD86 (r: 0.32), CD40 (r: −0.13), CD70 (r: 0.17), LAIR1 (r: 0.28), HAVCR2 (r: 0.33), and BTNL2 (r: −0.21) (**Figure 3H**). For patients from the GSE116918 (17), MDGPI was closely related to BTNL2 (r: −0.18), CD2 (r: 0.20), CD226 (r: 0.14), CD40 (r: −0.24), CD70 (r: −0.22), CD86 (r: 0.26), CD96 (r: 0.26), HAVCR2 (r: 0.31), ICOSLG (r: −0.23), LAG3 (r: −0.18), LAIR1 (r: 0.22), LAYN (r: 0.13), LGALS9 (r: 0.22), SIGLEC15 (r: −0.20), TIGIT (r: −0.17), TNFRSF18 (r: −0.26), TNFRSF8 (r: −0.21), TNFSF14 (r: −0.21), and VTCN1 (r: −0.19) (**Figure 3I**). Among the above genes (**Figures 3F–I**), patients who had higher expression of CD96 (HR: 1.79, 95% CI: 1.06–3.03) in the GSE116918 (17), and higher level of TNFSF18 (HR: 2.31, 95% CI: 1.38–3.86), CD80 (HR: 1.75, 95% CI: 1.05–2.93), and CD160 (HR: 2.22, 95% CI: 1.32–3.72) in the TCGA database were more prone to BCR than their counterparts (**Figure 3J**).

### Function and Drug Analysis

**Figure 4:**
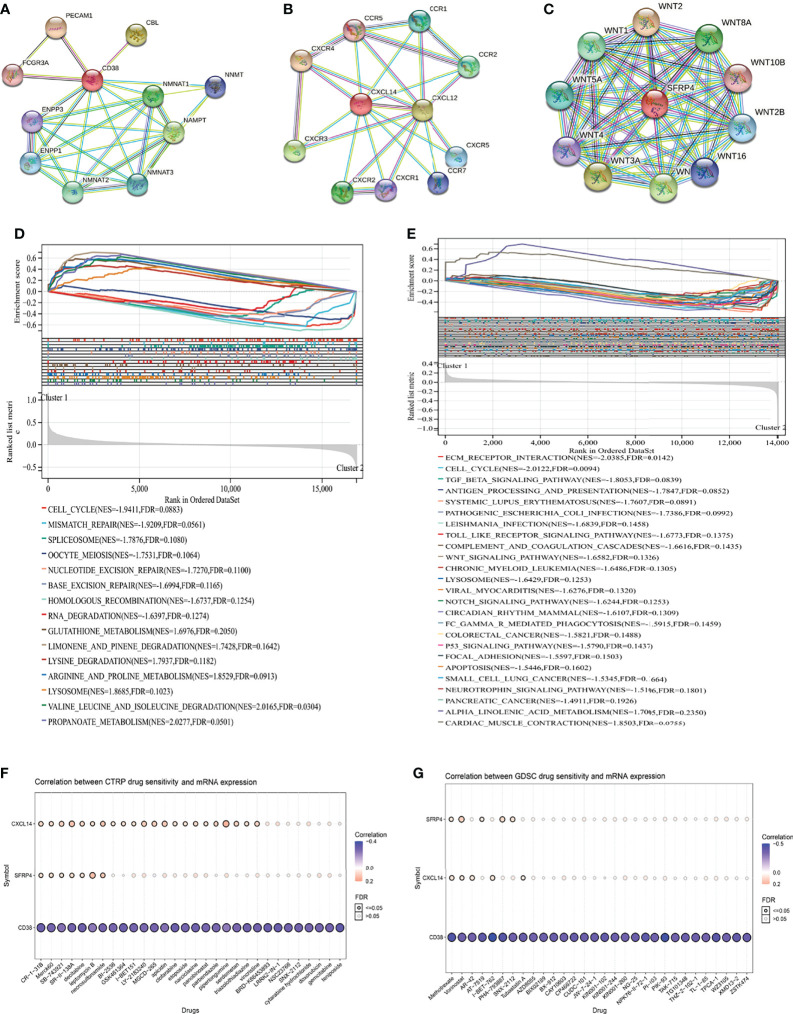
Function and drug analysis. **(A)** predicted functional partners of CD38; **(B)** predicted functional partners of CXCL14; **(C)** predicted functional partners of SFRP4; **(D)** GSEA analysis of the two clusters in the TCGA database; **(E)** GSEA analysis of the two clusters in the GSE116918 (17); **(F)** correlation between CTRP drug sensitivity and mRNA expression of CXCL14, CD38, and SFRP4; **(G)** correlation between GDSC drug sensitivity and mRNA expression of CXCL14, CD38, and SFRP4. GSEA, gene set enrichment analysis; CTRP, cancer therapeutics response portal; GDSC, genomics of drug sensitivity in cancer.

In order of the predicted scores from the highest to the lowest in the STRING database (22), the predicted functional partners of CD38 were PECAM1, CBL, NAMPT, NMNAT1, NMNAT2, NMNAT3, ENPP1, NNMT, ENPP3, and FCGR3A (**Figure 4A**); for CXCL14, the predicted interaction genes were CXCR4, CXCL12, CXCR3, CXCR2, CCR2, CCR1, CXCR5, CCR7, CXCR1, and CCR5 (**Figure 4B**); for SFRP4, the predicted interaction genes were WNT2, WNT3A, WNT7A, WNT8A, WNT1, WNT5A, WNT2B, WNT16, WNT4, and WNT10B (**Figure 4C**). For patients undergoing radical prostatectomy, the highly enriched pathways in cluster 2 were cell cycle, mismatch repair, spliceosome, oocyte meiosis, nucleotide excision repair, base excision repair, homologous recombination, and RNA degradation (**Figure 4D**). For patients undergoing radical radiotherapy, the highly enriched pathways in cluster 2 were extracellular matrix (ECM) receptor interaction, cell cycle, TGF beta signaling pathway, antigen processing and presentation, Toll like receptor signaling pathway, complement and coagulation cascades, Wnt signaling pathway, chronic myeloid leukemia, lysosome, Notch signaling pathway, circadian rhythm mammal, Fc gamma R-mediated phagocytosis, colorectal cancer, P53 signaling pathway, focal adhesion, apoptosis, small cell lung cancer, neurotrophin signaling pathway, and pancreatic cancer (**Figure 4E**).

In terms of drug analysis, CTRP drug sensitivity showed that CR-1-31B, Merck60, SB-743921, SR-II-138A, decitabine, leptomycin B, and necrosulfonamide were potentially sensitive to CXCL14, SFRP4, and CD38 (**Figure 4F**), while GDSC drug sensitivity showed that methotrexate and vorinostat were potentially sensitive to CXCL14, SFRP4, and CD38 (**Figure 4G**).

## Discussion

Although the 5-year relative survival rate of PCa is as high as 98% in America, its estimated death toll is second only to lung cancer and its long-term decline in cancer mortality since 1993 has stopped (1). The global population of individuals over 65 years old is growing rapidly and about 20% of the world population will be aged 65 or older by 2030 (28). PCa, as an aging-related cancer with a high incidence in men over 65 (1), will affirmatively attract considerable attention with an aging population worldwide. Mitochondria are highly evolved organelles that govern energy production, distribution and biosynthesis (29). MIDS is one of the typical phenotypes of aging, which could lead to reactive oxygen species-(ROS) driven lipid damage, deposits, and lipofuscin accumulation (6). Except for oxidation, lipid-derived aldehyde byproducts, such as 4-hydroxy-2-nonenal have been reported in senescent cells (6, 30). Furthermore, increased senescence has been observed in the mouse models of MIDS and elevated oxidative stress (31). Decreased NAD^+^/NADH ratios were observed in senescent cells, the possible mechanism was the accumulated pro-inflammatory M1-like macrophages in metabolic tissues during aging which expressed high level of NAD-consuming enzyme CD38 and thereby reduced tissue NAD levels (31, 32). Given the important role of MIDS in cellular senescence, it is reasonable to link MIDS to cellular senescence and senescence-related diseases, the most important of which are tumors. From the perspective of cancergenesis, MIDS may be the result of mutations in oncogenes and tumor suppressor genes, since changes in the expression levels of oncogenes and tumor suppressor genes such as TP53, which is discussed in next paragraph, and also bcl-2, HIF-1α may directly affect mitochondrial respiration and metabolism, resulting in MIDS (33, 34). In prostate, zinc in normal prostate epithelial cells slows the tricarboxylic acid cycle (TCA) and ATP production, whereas PCa cells observe TCA and ETC activation (35, 36). Despite cancergenesis, there is growing evidence that MIDS is associated with cancer cell survival, proliferation, recurrence and metastasis, the strongest evidence of which attributes to mitochondrial-derived ROS (mROS) (37). The binding of mitochondrial to SUMO-deficient hexokinase 2 (HK2) enhanced glucose consumption and lactate production, along with reduced mitochondrial respiration, resulted in PCa cell proliferation (38). Oxidative phosphorylation-related sulfite oxidase in mitochondria was found to be associated with BCR in post-prostatectomy patients, and the elevated Ki-67 LI score suggested that the mechanism of recurrence may be related to the activation of oxidative phosphorylation and the induction of cell proliferation (39). Porporato et al. proposed a hyper-invasive and hyper-metastatic tumor cell phenotype centered on multiple mitochondrial pathways, including ETC overload or partial ETC inhibition and increased succinate and superoxide production, with protein tyrosine kinases Src and Pyk2 as downstream effectors (40). Furthermore, understanding the relationship between MIDS and cancer also opens up therapeutic opportunities, for example, new evidence suggests that mitochondrial remodeling is critical in apoptosis and programmed death (41–43), suggesting that MIDS may be a marker for cancer detection and a target for treatment, especially radiation and chemotherapy, which achieves its therapeutic purpose by inducing apoptosis (44). Treatment with the mitochondria-specific superoxide scavenger mitoTEMPO can prevent metastasis *in vitro* (40). 5-(4-methoxyphenyl)-3H-1,2-dithiole-3-thione (AOL), a member of a new class of mROS inhibitors, reduces steady-state cellular ROS levels in human lung cancer cells, expressing anticancer properties (45). MIDS, while being a metabolic marker of cancer cells (46), establishes a pathway for drug resistance in tumor cells. For example, biguanides, tigecycline and gamitinib inhibit tumor cell energy synthesis by reducing the mitochondrial electron transport chain (ETC), but the upregulation of glycolytic genes compensates for the lack of ATP production, resulting in drug insensitivity (47–49). In addition, mitochondrial metabolism-related enzymes such as SUMO-deficient HK2 and mitochondrial 2,4-dienoyl-CoA reductase (DECR1) mentioned above also associated with resistance to docetaxel (38) and resistance to bicalutamide, apalutamide, or enzalutamide (50). Radioresistance is an adaptive response to radiation-induced damage by altering several cellular processes that sustain tumor growth. Mitochondria and metabolic reprogramming have been implicated in many cellular processes involved in radioresistance (51, 52). For example, enzymes important in base excision repair are localized to mitochondria or actively transported to mitochondria (53). In addition, cell cycle, oncogenes, tumor suppressor genes, autophagy, cellular metabolism, and ROS are also sites for mitochondria-mediated radioresistance (52). For PCa, lactate dehydrogenase A (LDHA), a major metabolic enzyme that produces lactate, is an enzyme that has been shown to be closely related to the glycolytic pathway and PCa radioresistance, and LDHA-targeted therapy combined with radiotherapy can improve the radiosensitivity of radioresistant PCa cells (54). It is clear that PCa undergoes metabolic reprogramming, in this process, MIDS is indeed cross-linked with the occurrence, development, recurrence, metastasis and treatment resistance.

Pelicano et al. (55) found that MIDS and ROS imbalance promoted breast cell motility and the mechanism was that overexpressed CXCL14 could cell motility through elevation of cytosolic Ca (2+) by binding to the inositol 1,4,5-trisphosphate receptor on the endoplasmic reticulum. Besides, SFRP4 was an important Wnt signaling antagonist, and activation of SFRP4 could lead to Wnt signaling suppression and histone modification in PCa stem cells and thereby sensitized tumor cells to chemotherapeutic drugs, enhancing cell death (56). The decrease in mitochondrial ATP could reduce calcium uptake into the endoplasmic reticulum, leading to endoplasmic reticulum stress and to impaired Wnt signaling; in turn, the recovery of the ATP level or the inhibition of endoplasmic reticulum stress restored Wnt activity (57). Thus, CXCL14, CD38, and SFRP4 were closely associated with MIDS. In this study, using the above three genes, we firstly proposed distinct prognosis-related molecular subtypes from the fresh perspective of MIDS for PCa patients undergoing radical prostatectomy or radiotherapy. Moreover, the molecular subtype was highly associated with the T stage and Gleason score, both of which were closely related to the prognosis of PCa (58). In addition, this classification was an independent risk factor for patients undergoing radical radiotherapy.

It is worth noting that we have observed different or even opposite results for the infiltrated immune cells under the two different treatments for PCa patients. This requires us to critically look at the role of inflammation in PCa progression. For patients undergoing radical radiotherapy, various immune cells, namely, CD8^+^ T cells, neutrophils, macrophages, and dendritic cells are more enriched in cluster 2, the group with a worse prognosis, and the tumor purity is lower. Besides, MDGPI had highly positive correlations with these immune cells. Radiotherapy can trigger and induce inflammation/immune response through factors such as DNA damage, cell death and senescence, immune cell response, cellular stress, hypoxia, and tumor antibodies (59). Oxidative stress and DNA damage caused by radical radiotherapy are both considered the initiation events of PCa (60, 61). Furthermore, for PCa patients with severe inflammation, it was observed that their recurrence-free survival was shorter (62). In addition, Schoenfeld et al. found that the single nucleotide polymorphisms of RNASEL, a gene implicated in inflammation, significantly reduces the risk of BCR in patients with radical radiotherapy, but no significant impact on patients with radical prostatectomy (63). Research has observed that increased T cell density has been associated with PTEN loss and poorer outcome in African American men with PCa, with lower BCR-free survival, which is partly consistent with our research results (64, 65). This also explains why in our study, the stromal score and immune score are higher, and cluster 2 with lower tumor purity has a worse prognosis for PCa patients after radical radiotherapy. However, for patients undergoing radical prostatectomy, we did not observe any statistically significant results except for CD8^+^ T cells between cluster 2 and cluster 1, and a negative correlation between MDGPI and CD8^+^ T cells was detected. CD8^+^ T cells are the most powerful effectors in the anticancer immune response (66), and the reduced CD8^+^ T cells might contribute to the worse prognosis of cluster 2 undergoing radical prostatectomy. An interesting finding was the completely opposite trends in CD8^+^ T cells between radical prostatectomy and radical radiotherapy groups. For radical prostatectomy, the poor prognosis group (cluster 2) showed low levels of CD8^+^ T cells, CD8^+^ T cells were higher in the radiotherapy group. CD8^+^ effector cells are the main effector cells for targeting antitumor immune response (67). In the radical prostatectomy patient population, low levels of CD8^+^ T cells may predict clearance of tumor antigens and low antitumor immune responses, leading to fewer recurrences and metastases. Studies have found that the proportion of CD8^+^ cells transiently decreased after the first four weeks of radiotherapy, while the proliferation rate of CD8^+^ T cells increases at the end of radical radiotherapy for PCa and persists until three months after treatment, and the frequency and function of antigen-specific CD8^+^ T cells remained stable during treatment (68). Radiation has proinflammatory and immunomodulatory effects and, contrary to popular belief, promotes antitumor immune responses, namely, T cell homing and tumor infiltration (69, 70). Lin et al. found that PD-1 expression began to increase after chemotherapy-induced increases in the number of CD8^+^ tumor-infiltrating lymphocytes immediately after radiotherapy, suggesting that these CD8^+^ T cells began to become functionally exhausted (71). In addition, we observed a higher positive correlation between MDGPI and stromal score, which indicated the role of stromal components in the progression of PCa patients undergoing radical prostatectomy. Reactive stroma has been used to assess the PCa-specific mortality in diagnostic prostate needle biopsies (72). TNFRSF18, CD86, CD40, CD70, LAIR1, HAVCR2, and BTNL2 were common checkpoints which were differentially expressed between the two clusters and associated with MDGPI for patients undergoing radical prostatectomy or radiotherapy. However, TNFRSF18 and CD70 showed opposite results after receiving the different treatments, both of which seemed to be involved in interactions between activated T-lymphocytes (73). The paradoxical results of the two genes could partially explain the progression mechanism of PCa patients undergoing different treatment when combined the opposite results of immune-infiltrating cells. Furthermore, CD96, TNFSF18, CD80, and CD160 identified in this study might be the potential targets of PCa due to their prognostic values. CD96 may play a role in the adhesion interaction between activated T cells and NK cells in the late immune response. According to Biograph’s knowledge base, in the context of CD96, PCa ranks 4th among 6021 disease concepts, strongly indicating the potential role of this gene in the development of PCa (74). The expression of T-cell immunoglobulin domain and mucin domain-containing molecule 3 (TIM-3) encoded by HAVCR2 in CD4^+^ and CD8^+^ T cells of the PCa patients was significantly increased compared with benign prostatic hyperplasia, suggesting that it may affect the development and progression of PCa (75). In addition, TIM-3 expression is also related to the poor prognosis of PCa (76, 77). In the TCGA group, CD80 mRNA expression is correlated with BCR, suggesting that genetic variation and mRNA expression in CD80 may be a predictor and potential target of local PCa (78). In fact, several clinical studies targeting CD80 (B7-1) have approved, for example, ipilimumab, an antagonistic monoclonal antibody that binds CD80 on antigen-presenting cells, providing options for PCa patients in the future (79). The findings of these immune checkpoints are helpful to the choice of medications for adjuvant treatment of patients undergoing radical prostatectomy or radiotherapy.

GSEA analysis shows that the results of our research are related to a variety of cancers, such as colorectal cancer, small cell lung cancer, etc., which further proves the clinical significance of our molecular subtypes. In addition, it was also found to be related to the p53 signaling pathway. Li et al. found that p53-mediated mitochondrial dysfunction can promote PCa cell apoptosis *in vitro* (80). In addition, abnormal activation of Notch signal has also been shown to be closely related to the occurrence and development of PCa (81). The deletion of TP53, a tumor suppressor gene, can regulate mitochondrial respiration by promoting the Warburg effect in cancer cells, while increase the uptake of glucose in cancer cells through the repression of transcription of glucose transporter (GLUT) isoforms 1 and 4 and inhibition of the expression of glycolytic enzymes (82–85).TP53 gene improves the fidelity of DNA replication and homologous recombination through transcriptional activation of mismatch repair (MMR) genes. Abnormal MMR protein expression may be involved in the progression of PCa (86). In addition, cluster 2 was found to be related to lysosome in both TCGA and GEO groups. Lysosomes are known to be involved in a variety of cancer processes. Various risk factors for PCa, such as ionizing radiation and oxidative stress, can activate the activity of lysosomal enzymes, which may cause cancer to occur by destroying proteins and other components of cells (87). Meanwhile, abiraterone can inhibit the proliferation of PCa cells *in vitro*, thus promoting apoptosis by regulating mitochondrial autophagy (88). Basic autophagy genes are ubiquitous in tumors including PCa, and autophagy defects promote tumorigenesis (89). On the other hand, tumor cells are also dependent on autophagy, and the loss of autophagy gene inhibits the formation and metastasis of the primary tumor (90, 91). In this study, we also found some sensitive drugs to the investigated three genes, which needed to be further studied *in vivo* and *in vitro*.

As the aging of the global population continues to develop in the coming decades, PCa in elderly men will bring a huge burden of disease. At present, there is no optimal plan for the management of BCR. In this paper, we calculated that the gene prognostic index composed of CXCL14, SFRP4, and CD38, can well predict the pathogenesis of individual patients with PCa after radical prostatectomy and radiotherapy. In this way, from a clinical perspective, a timely warning can be given before the thorny problem of insufficient treatment methods and poor prognosis for BCR and metastasis patients. At the same time, the discovery of the three targeted genes also avoided tedious and expensive whole-genome sequencing. Our research integrates two high-throughput sequencing and microarray sequencing platforms, as mutual verification, the results are more reliable and have strong clinical relevance.

### Conclusions

We proposed distinctly prognosis-related molecular subtypes at genetic level and related formula for PCa patients undergoing radical prostatectomy or radiotherapy, mainly to provide a roadmap for precision medicine.

## Data Availability Statement

The original contributions presented in the study are included in the article/**Supplementary Material**. Further inquiries can be directed to the corresponding authors.

## Author Contributions

DCF proposed the project, conducted data analysis, interpreted the data, and wrote the manuscript. XS, FCZ, and QX conducted data analysis, interpreted the data. QW and LY supervised the project, and interpreted the data. All authors listed have made a substantial, direct, and intellectual contribution to the work and approved it for publication.

## Funding

This program was supported by the National Natural Science Foundation of China (Grant Nos. 81974099, 82170785, 81974098, 82170784), the programs from the Science and Technology Department of Sichuan Province (Grant No. 21GJHZ0246), the Young Investigator Award of Sichuan University 2017 (Grant No. 2017SCU04A17), the Technology Innovation Research and Development Project of Chengdu Science and Technology Bureau (2019-YF05-00296-SN), and the Sichuan University—Panzhihua Science and Technology Cooperation Special Fund (2020CDPZH-4). The funders had no role in study design, data collection or analysis, preparation of the manuscript, or the decision to publish.

## Conflict of Interest

The authors declare that the research was conducted in the absence of any commercial or financial relationships that could be construed as a potential conflict of interest.

## Publisher’s Note

All claims expressed in this article are solely those of the authors and do not necessarily represent those of their affiliated organizations, or those of the publisher, the editors and the reviewers. Any product that may be evaluated in this article, or claim that may be made by its manufacturer, is not guaranteed or endorsed by the publisher.

## Abbreviations

PCa, Prostate cancer; BCR, biochemical recurrence; SASP, senescence-associated secretory phenotype; ROS, reactive oxygen species; MMR, mismatch repair; TIM-3, T-cell immunoglobulin domain and mucin domain-containing molecule 3; TME, tumor immune microenvironment; MIDS, mitochondrial dysfunction; DEGs, Differentially expressed genes; GEO, Gene Expression Omnibus; CTRP, cancer therapeutics response portal; GDSC, genomics of drug sensitivity in cancer; GSEA, Gene set enrichment analysis; FDR, false discovery rate; MDGPI, MIDS-related genetic prognostic index.
