# Supplementary material for: Mitochondria Dysfunction-Mediated Molecular Subtypes and Gene Prognostic Index for Prostate Cancer Patients Undergoing Radical Prostatectomy or Radiotherapy
Source: Front Oncol. 2022 Apr 6;12:858479. doi: 10.3389/fonc.2022.858479 (PMC9019359; doi:10.3389/fonc.2022.858479)
Supplement: Supplementary file 1 [file DataSheet_1.docx]

Supplementary figure 1. The process of merging four GEO datasets.


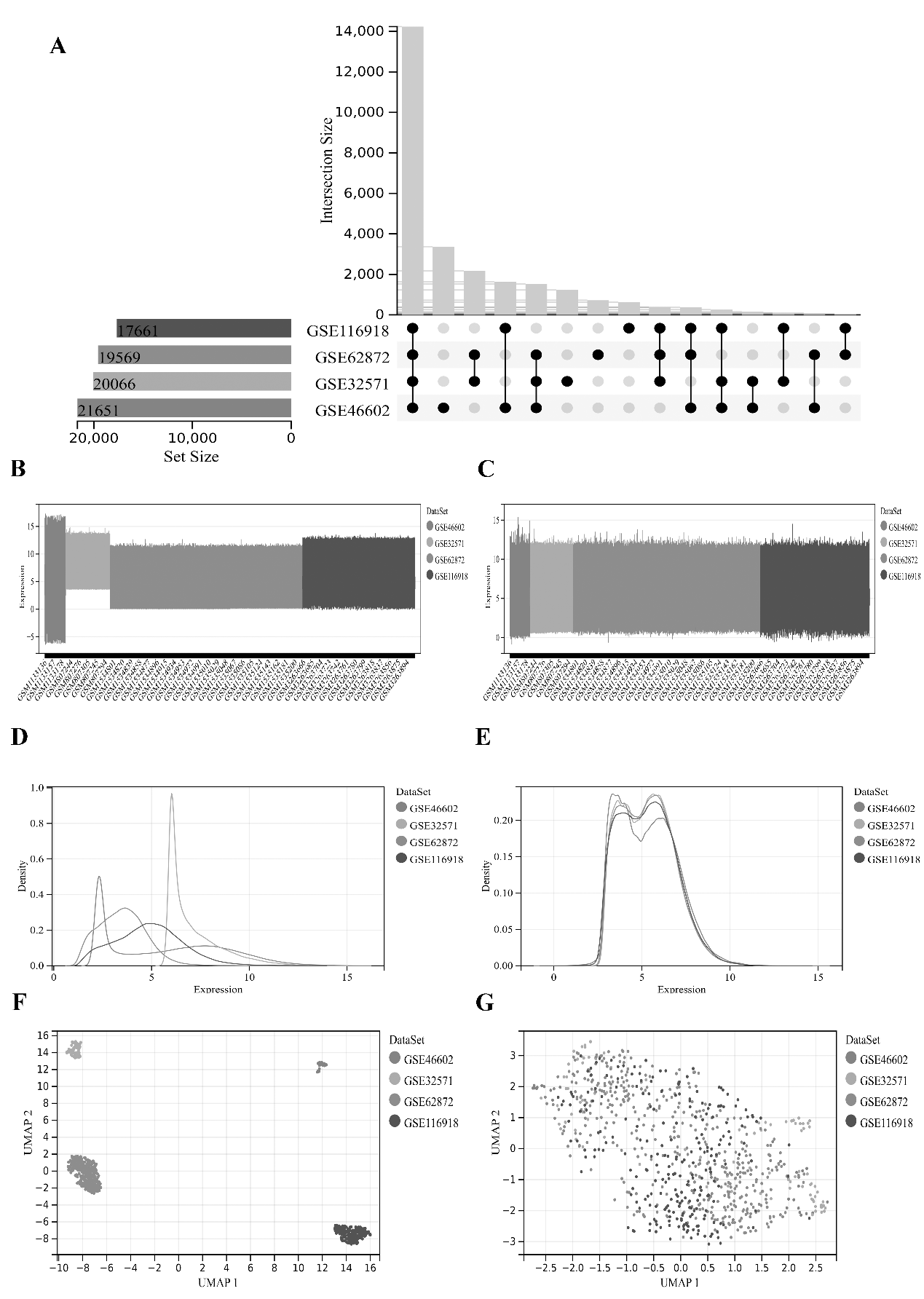


Supplementary figure 1. The process of merging four GEO datasets. (A) upset plot; (B) box plot before removing batch effects; (C) box plot after removing batch effects; (D) density plot before removing batch effects; (E) density plot after removing batch effects; (F) umap plot before removing batch effects; (G) umap plot after removing batch effects. GEO= Gene Expression Omnibus.
